# Amaryllidaceae alkaloids: identification and partial characterization of montanine production in *Rhodophiala bifida* plant

**DOI:** 10.1038/s41598-019-44746-7

**Published:** 2019-06-11

**Authors:** Andressa Reis, Kevin Magne, Sophie Massot, Luciana R. Tallini, Marina Scopel, Jaume Bastida, Pascal Ratet, José A. S. Zuanazzi

**Affiliations:** 1 Laboratory of Pharmacognosy, Department of Raw Material Production, Federal University of Rio Grande do Sul, 90610-000 Porto Alegre, UFRGS Brazil; 2 Institute of Plant Sciences Paris-Saclay IPS2, CNRS, INRA, Université Paris-Sud, Université Evry, Université Paris-Saclay, Bâtiment 630, 91405 Orsay, France; 3 Institute of Plant Sciences Paris-Saclay IPS2, Paris Diderot, Sorbonne Paris-Cité, Bâtiment 630, 91405 Orsay, France; 4 Natural Products Group, Faculty of Pharmacy, University of Barcelona, Av. Joan XXIII, 27-31, 08028 Barcelona, Spain; 5 Present Address: Laboratory of Molecular Biology, Department of Plant Sciences, Wageningen University & Research, 6708 PB Wageningen, The Netherlands

**Keywords:** DNA, RNA sequencing, Secondary metabolism

## Abstract

*Rhodophiala bifida* (*R*. *bifida*) is a representative of the Amaryllidaceae plant family and is rich in montanine, an alkaloid with high pharmaceutical potential. Despite the interest in these compounds, many steps of the biosynthetic pathway have not been elucidated. In this study, we identified the alkaloids produced in different organs of *R*. *bifida* under different growth conditions, set up the conditions for *in vitro R*. *bifida* regeneration and initiated the molecular characterization of two *R*. *bifida* genes involved in alkaloids biosynthesis: the *Norbelladine 4*′*-O-Methyltransferase* (*RbN4OMT*) and the *Cytochrome P450* (*RbCYP96T*). We show that montanine is the main alkaloid produced in the different *R*. *bifida* organs and developed a direct organogenesis regeneration protocol, using twin-scale explants cultivated on media enriched with naphthalene acetic acid and benzyladenine. Finally, we analyzed the *RbN4OMT* and *RbCYP96T* gene expressions in different organs and culture conditions and compared them to alkaloid production. In different organs of *R*. *bifida* young, adult and regenerated plants, as well as under various growing conditions, the transcripts accumulation was correlated with the production of alkaloids. This work provides new tools to improve the production of this important pharmaceutical compound and for future biotechnological studies.

## Introduction

Plants belonging to the Amaryllidaceae family produce alkaloids that have been extensively studied because of their pharmaceutical properties. The genus *Rhodophiala* (Amaryllidaceae) is endemic in South America and contains more than 30 bulbous species showing ornamental potential due to their colorful and attractive flowers. These plants usually grow in restricted areas and are geographically isolated^1^. Amaryllidaceae alkaloids such as galanthamine present anticholinesterase activities used for the treatment of Alzheimer’s disease; lycorine presents cytotoxicity and antitumor properties. Recently, it was shown that montanine has anxiolytic, antidepressive and anticonvulsive activities as well as immunomodulatory properties. Moreover, montanine has acetylcholinesterase inhibition, anti-reumatic, antimicrobial and antiproliferative effects. These important pharmaceutical properties justify an increasing of interest towards this class of compounds^2–10^.

Despite all the pharmaceutical interests of the galanthamine molecule and the search for new drugs derived from the Amaryllidaceae alkaloids (AmAl), the corresponding biosynthetic pathway is not fully characterized yet. Enzymatic steps remain partially misunderstood, especially for the montanine biosynthesis pathway which is distinct from the galanthamine one. By contrast, montanine is characterized by a unique 5,11-methanomorphanthridine skeleton^11^.

**Figure 1:**
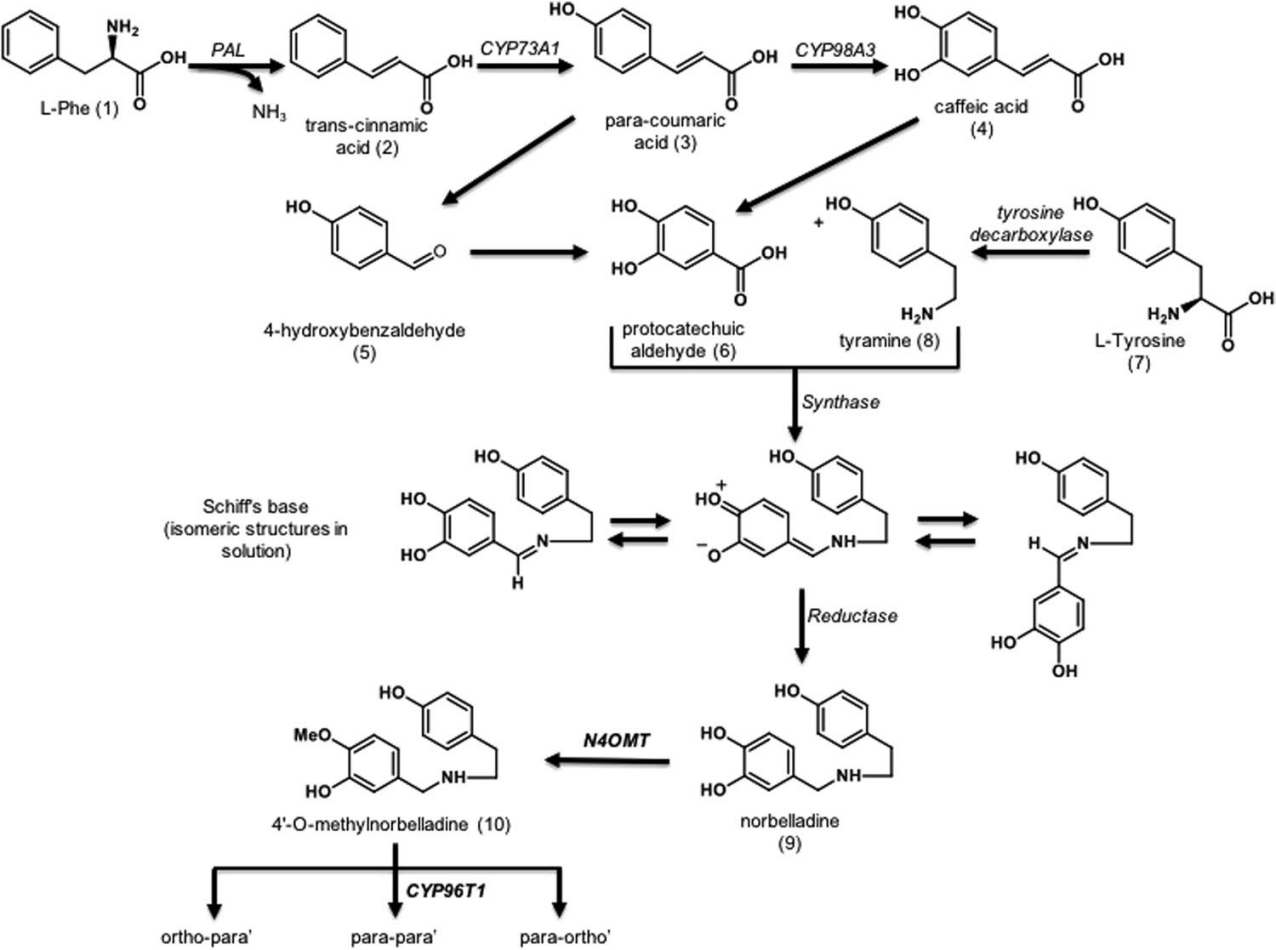
The Amaryllidaceae alkaloid biosynthetic pathway: The AmAl precursor 4′-*O*-methylnorbelladine biosynthetic pathway^14^. Abrev.: L-Phe (L-phenylalanine), N4OMT (norbelladine 4′-*O*-methyltransferase) and CYP96T1 (Cytochrome P450 enzyme of 4′-*O*-methylnorbelladine).

AmAl biosynthesis (Fig. 1) starts from an L-phenylalanine (**1**). The Phenylalanine ammonia-lyase (PAL) enzyme generates *trans*-cinnamic acid (**2**). The CYP73A1 cytochrome P450 enzyme then produces *p*-coumaric acid (**3**) which forms 4-hydroxycinnamic acid (caffeic acid) (**4**) or 4-hydroxybenzaldehyde (**5**) through the action of the CYP98A3. Both molecules can generate protocatechuic aldehyde (**6**). Caffeic acid is transformed by the action of a Vanillin synthase (*Vp*VAN) homolog^12–14^. Tyrosine decarboxylase transforms L-tyrosine (**7**) in tyramine (**8**). Tyramine can then be condensed together with protocatechuic aldehyde (**6**) by an unknown enzyme to form a Schiff-base that will be reduced by a reductase to form norbelladine (**9**). Norbelladine is then methylated by Norbelladine 4′-*O*-Methyltransferase (N4OMT) to form 4′-*O*-methylnorbelladine (**10**)^14^.

**Figure 2:**
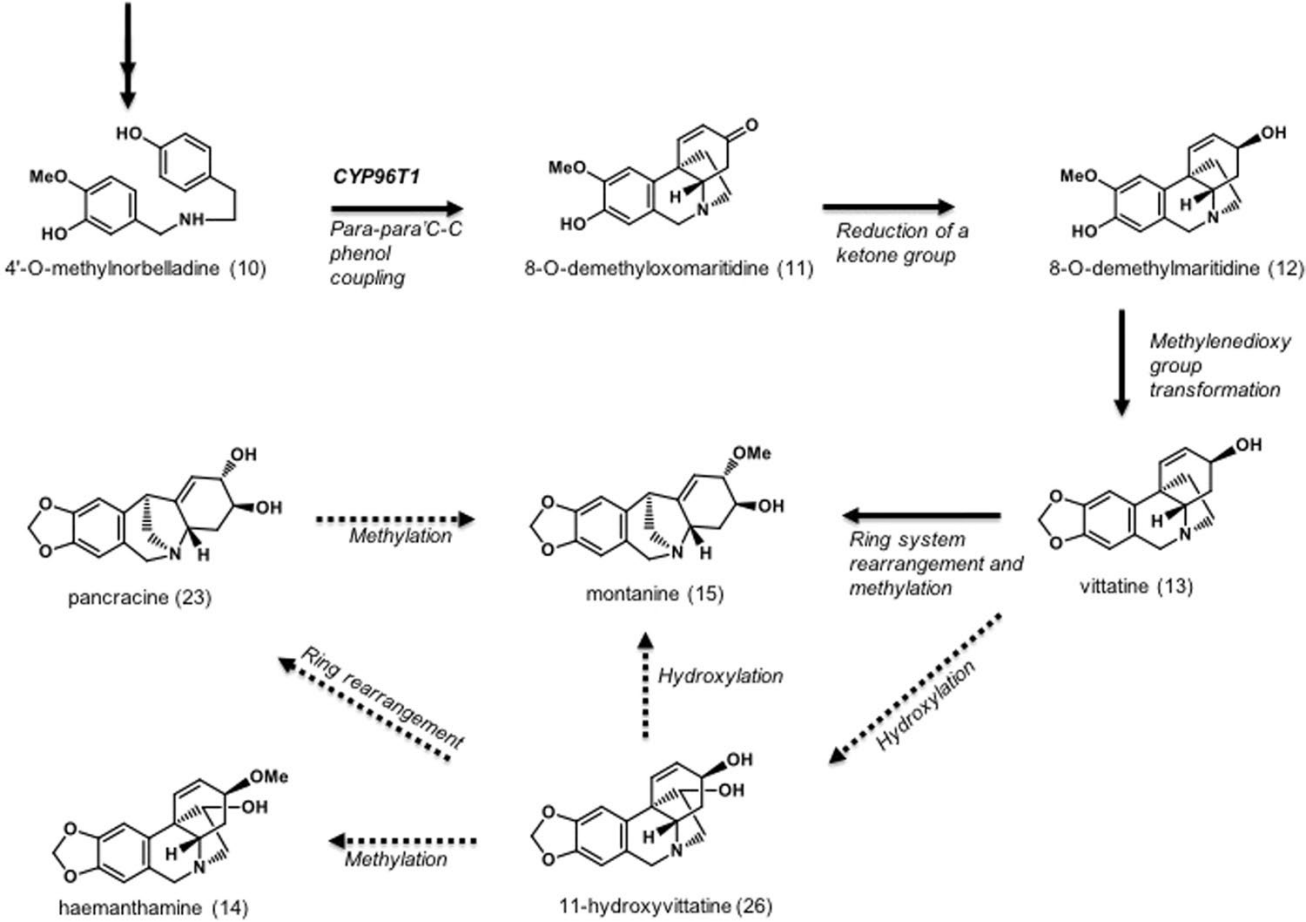
The montanine biosynthetic pathway: Schematic representation showing the important steps of the montanine-type alkaloid formation in Amaryllidaceae plants^18,19,21–23^.

The key intermediate step for the multiple biosynthetic pathways producing the various structural types of AmAl (Fig. 2) is the 4′-*O*-methylnorbelladine cyclization by the CYP96T Cytochrome P450 enzyme. This cyclization consists in phenol-phenol oxidative coupling and requires *ortho-para’*, *para-para’* and *para-ortho’* C-C coupling^14–17^. The 8-*O*-demethyloxomaritidine (**11**) is formed following *para-para’* C-C phenol coupling. This compound is a dienone intermediate precursor of the alkaloid’s skeleton types such as haemanthamine, tazettine, crinine, narciclasine and montanine. After a reduction of the ketone group, 8-*O*-demethylmaritidine (**12**) is formed and is a likely precursor of vittatine (**13**) via methylenedioxy group transformation^12,16,18,19^.

Studies with *R*. *bifida* plants^15,20^ indicate that 11-hydroxyvittatine (**26**) (haemanthamine ring system) is formed by the addition of a hydroxyl group to the vittatine (**13**). This molecule might be both the montanine and haemanthamine precursors, with biosynthesis of haemanthamine being more efficient (only a methylation of the hydroxyl function in C_3_ of 11-hydroxyvittatine). For montanine (**15**) formation the rearrangement of the vittatine ring system is necessary to promote the methylation of the oxygen in C_2_^21–23^.

Genes involved in the AmAl biosynthesis are poorly characterized and transcriptomic studies as well as genome sequencing were only recently initiated for this plant family^24–26^. The class I *O*-methyltransferase *Norbelladine 4*′*-O-Methyltransferase* (*N4OMT*) gene was recently identified in *Narcissus* sp. *aff*. *pseudonarcissus* by heterologous expression in *E*. *coli*^27^. Also, the gene encoding Cytochrome P450 *CYP96T1* responsible for the *para-para’* phenol coupling reaction generating both haemanthamine and crinine carbon skeletons was characterized in *Narcissus*^28^.

Because of the high biotechnological interest for the montanine produced by *R*. *bifida*, we identified the alkaloids produced in this plant, set up *in vitro R*. *bifida* regeneration conditions and cloned the *RbN4OMT* and *RbCYP96T* genes. The gene expression of *RbN4OMT* and *RbCYP96T* were analyzed and correlated with alkaloids production in different organs of *R*. *bifida* young, adult and regenerated plants under different culture conditions. This work sets up the bases for further studies aiming at improving the montanine production.

## Results

### Montanine is the major alkaloid produced in *R*. *bifida*

The amount of the different types of alkaloids produced in *R*. *bifida* bulbs, leaves and roots were evaluated using GC-MS analysis (Table 1). Montanine, possesses a 5,11-methanomorphanthridine nucleus and differs from the other montanine type alkaloids in the substituents at C-2 and C-3^29^. According to our study, montanine (**15**) was present in all organs and is the most abundant alkaloid of the plant, in a range from 49.7 to 88% of Total Ion Current (TIC, Table 1). The highest amount was found in roots (74–88% of TIC) and the lowest in leaves (54–56% of TIC). Another study using *R*. *bifida* bulbs, indicated that the alkaloid extracts contained 92% of montanine^2^ and only traces (less than 0.20%) of vittatine, deoxytazettine, tazettine, pretazettine and 3-epimacronine. In contrast, in our study the amount of tazettine (**22**) was in a range of 1.3 to 13.1% of TIC, with the lowest amount in bulbs (1.3 to 2.9% of TIC) and the highest amount in leaves (13.1% of TIC). This compound is a part of the tazettine-type alkaloids, derived from the 2-benzopyrano[3,4-c] indole skeletons, widely reported in *Narcissus* and known as an extraction artifact of pretazettine^29^. Vittatine (or crinine, **13**) is an intermediate in narciclasine synthesis and is thus involved in the montanine formation. It was found in small concentrations in bulbs and leaves^29^. Pancracine (**23**) was the other molecule identified in all the analyzed tissues. It belongs to the montanine-type alkaloids identified in the 60’s in *Pancratium maritimum*, *Narcissus poeticus* and *R*. *bifida*^30^. The amount of this compound ranged from <0.1 to 13% of TIC, with the lowest yield in roots (<0.1–4.3%) and bulbs (1.3–3.3%) and higher content in leaves with 11.8 to 13% of TIC. We believe that the differences with previous studies^2^ may result from different harvesting times, places, soil, variety or from different chemotypes related to the species sampled.

**Table 1:** *R*. *bifida* alkaloids identified by GC-MS analysis.

Compound	Rt (min)	RI	[M]^+^	Bulbs	Leaves	Roots
Ismine (16**)	20.61	2267.7	257	<0.1–1.0	<0.1–1.0	<0.1
Trisphaeridine (**17**)	20.92	2286.6	223	<0.1	<0.1	<0.1
5,6-Dihydrobicolorine (**18**)	21.62	2330.1	239	<0.1	<0.1	—
*m/z* 270^*a^; [M = 343]^*b^, galanthamine-type^*c^	23.57	2454.5	343	—	—	<0.1
*m/z* 238^*a^; [M = 301]^*b^, narciclasine-type^*c^	23.59	2455.2	301	0.6–1.0	1.2	<0.1
*m/z* 288^*a^; [M = 289]^*b^, galanthamine-type^*c^	23.70	2463.0	289	—	<0.1	—
Vittatine/Crinine (**13**)	23.89	2475.9	271	1.0–1.22	<0.1	—
Galanthindole (**20**)	24.23	2498.3	281	<0.1–2.1	<0.1–0.8	—
8-*O*-Demethylmaritidine (**12**)	24.40	2509.6	273	<0.1	—	—
*O*-Methyltazettine (**21**)	25.81	2604.7	345	<0.1–3.3	<0.1	—
Montanine (**15**)	26.36	2641.4	301	49.7–77.6	53.7–56.3	74–88.0
Tazettine (**22**)	26.52	2652.4	331	1.3–2.9	13.1	8.5–9.3
*m/z* 252^*a^; [M = 343]^*b^, lycorine-type^*c^	26.61	2658.2	343	—	11.8	—
*m/z* 331^*a^; [M = 331]^*b^	26.70	2664.4	331	<0.1	—	—
Pancracine (**23**)	27.21	2698.0	287	1.3–3.3	11.8–13.0	<0.1–4.3
3-*O*-Acetylpancracine (**24**)	27.77	2736.1	329	—	1.6	—
*m/z* 270^*a^; [M = 329]^*b^, galanthamine-type^*c^	28.42	2779.3	329	—	<0.1	—
3-Epimacronine (**25**)	28.84	2807.6	329	<0.1	<0.1–1.1	<0.1
*m/z* 252^*a^; [M = 369]^*b^, lycorine-type^*c^	29.44	2848.0	369	—	<0.1	—
*m/z* 252^*a^; [M = 369]^*b^, lycorine-type^*c^	29.67	2863.1	369	—	4.1–5.0	—
*m/z* 252^*a^; [M = 387]^*b^, lycorine-type^*c^	31.07	2957.3	387	1.1–2.6	7.2–10.3	<0.1

^*a^Base peak. ^*b^Possible molecular ion peak. ^*c^Proposed structure-type according to the fragmentation pattern. **The molecules represented by bold numbers in brackets are in the biosynthesis pathways (Figs 1 and 2) or in the online resource, Fig. S3). *R*. *bifida* alkaloids detected by GC-MS analysis are given as percentage of TIC. TIC (for Total Ion Current) represents the amount of the different compounds detected by GC–MS in the different organs and expressed as a percentage of the total alkaloids present in *R*. *bifida* wild plants grown in a greenhouse. The identification of the alkaloid structural types was done by comparing their GC-MS spectra and Kovats Retention Index (RI) values with those of authentic AmAls previously isolated and identified by spectrometric methods (NMR, UV, CD, MS). The MS spectra were deconvoluted by AMDIS 2.64 software (NIST). Three samples were analyzed for each organ. Values are indicated as the proportion of each individual molecule in the alkaloid fractions.

The 8-*O*-demethylmaritidine was detected in the genus *Galanthus*^31^ and as trisphaeridine (**17**), 5,6-dihydrobicolorine (**18**), and other traces of unknown molecules were found in our study. The molecule 5,6-dihydrobicolorine (**18**) was identified for the first time in *Narcissus bicolor* and belongs to the phenanthridine type alkaloids^32,33^. Ismine (**16**) and trisphaeridine (**17**) are considered as catabolic products from the haemanthamine- type skeleton^29^. Galanthindole (**20**) contains a non-fused indole ring and might represent an artifact of homolycorine or tazettine-type derivatives^34^. *O*-methyltazettine (**21**), 3-*O*-acetylpancracine (**24**) which possess a montanine type nucleus derived from pancracine^30^ and 3-epimacronine (**25**) identified in *Sprekelia formossisima* in the 60’s^35^ were detected as traces in our study.

Our analysis shows that montanine was the major alkaloid produced in *R*. *bifida* and that other molecules were found in low amount. For some of them we could identify only the alkaloid type and only one of them could not be identified (Table 1).

### *In vitro R*. *bifida* micropropagation

To initiate the *in vitro* culture of wild *R*. *bifida* plants we first tested different sterilization protocols. The disinfection method of the wild plants that used the pre-disinfection with sodium hypochlorite 8% allowed the lowest level of contamination and the establishment of about 84% healthy *in vitro* explants (Table 2). This pre-cleaning procedure provided a large reduction of the contamination by probably letting the bulbs drying on the outside and to be stored with a reduced number of microorganisms on their surface. This allowed subsequent better disinfection and production of healthy explants.

**Table 2:** Percentage of contamination in *R*. *bifida* bulbs explants using different methods of disinfection.

Method	Contamination (%)
Sodium hypochlorite 2%	96.67 a
Heating at 54 °C	78.33 b
Pre-disinfection + Sodium hypochlorite 8%	16.00 c

Means with different letters are significantly different using Duncan test (*p* < 0.05).

The procedure using heat promoted the death of the *R*. *bifida* explants, in contrast to the results obtained with *Narcissus* ‘Golden Harvest’^36^ where the lowest percentage of contamination was obtained using hot water. For *Narcissus*, the treatment decreased contamination from 60% to only 5% without affecting the regeneration neither the vitality of explants. Factors related to bulb harvest in different places and seasons of the year as well as bulb size may make the surface disinfection of the plant highly variable^37^.

The use of different concentrations of growth regulators (GR) can control plant tissue proliferation and regeneration through cell division, elongation, differentiation and organogenesis. In addition they can modify secondary metabolites biosynthesis^15^. *R*. *bifida* calli, shoots and bulbs cultures were established *in vitro* by testing diverse GR contents (Table 3). GR promoted callogenesis at different levels, generating diverse types of calli, from friable to dense, but without regeneration capacity for the majority of them. Some of these media also allow explants to produce shoots by direct organogenesis or from calli, depending on the growth regulators used.

**Table 3:** Effects of different media on the callus and shoot formation percentage of *R*. *bifida* explants after 20 and 60 days of treatment.

Medium	Calli (20 days)	Calli (60 days)	Shoots (20 days)	Shoots (60 days)
1 (Sucrose 30 g/L)	32 bc	20 b	0 a	8 bc
2 (Sucrose 60 g/L)	12 bc	7 b	8 a	25 ab
3 (Sucrose 90 g/L)	40 b	0 b	0 a	4 bc
4 (NAA 5 mg/L + KIN 0.5 mg/L + BA 1 mg/L)	100 a	77 a	0 a	0 c
5 (2,4-D 1 mg/L + BA 1 mg/L)	88 a	76 a	0 a	0 c
6 (NAA 2 mg/L + BA 4 mg/L)	79 a	100 a	0 a	8 bc
7 (NAA 0.1 mg/L + BA 0.5 mg/L)	44 b	69 a	8 a	31 a
8 (NAA 1 mg/L + BA 2 mg/L)	100 a	78 a	0 a	7 bc
9 (NAA 0.1 mg/L + BA 5 mg/L)	0 c	0 b	0 a	20 abc
10 (NAA 2.5 mg/L + KIN 0.5 mg/L)	100 a	100 a	0 a	0 c

Means with different letters in the same column are significantly different using Duncan test (*p* < 0.05).

After 20 days of culture on medium 4, 5 and 8 calli formed on 100, 88 and 100% of explants, respectively. After 60 days, due to tissue death, callus formation declined to 77, 76 and 78%, respectively (Table 3). After 20 days of culture on medium 6, calli formed on 79% of the explants, and at 60 days, this percentage increased, reaching the totality of the explants. However, the best results, reaching 100% callogenesis, after both 20 and 60 days, were found using the medium 10 (Table 3), containing auxin and cytokinin, as growth regulators.

For axillary bud development, there were no significant statistical differences after 20 days of treatment, but at 60 days, the best result was obtained using medium 7, with 31% of explants forming buds. Using media 2 and 9, 25 and 20% of the explants formed buds (Table 3). These buds could latter develop to shoots and form micro-plants.

**Figure 3:**
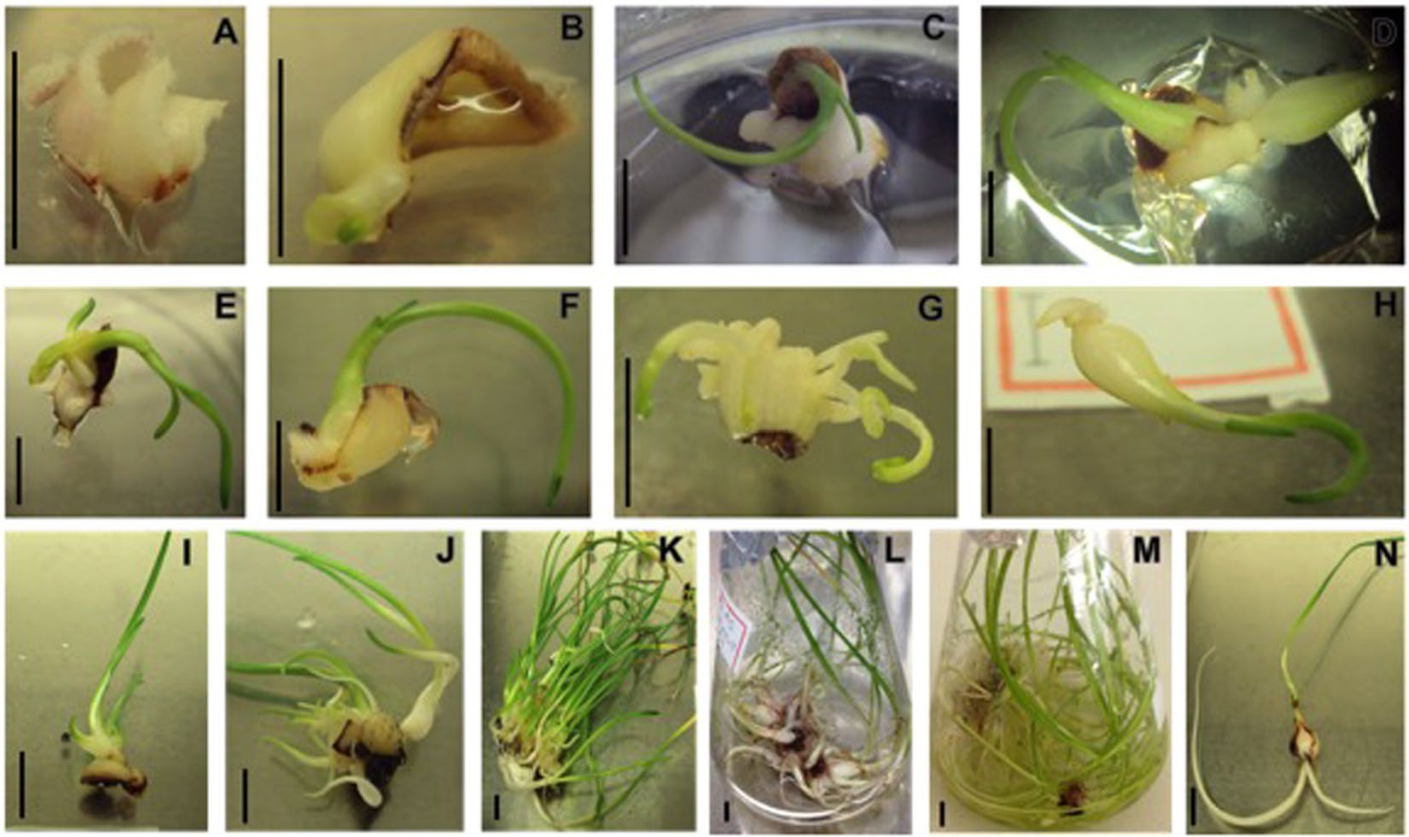
*In vitro R*. *bifida* regeneration: *R*. *bifida* regeneration process using twin-scales bulb explants. (**A**) Callus formation in control medium (sucrose 30 g/L); (**B**) Direct organogenesis in medium with sucrose 60 g/L; (**C**–**G**) Direct organogenesis and shoot regeneration in medium 7 (Medium 7: NAA 0.1 mg/L + BA 0.5 mg/L); (**H**) Bulb and root formation; (**I**) Shoots growing attached to the explant; (**J**) Shoot multiplication and bulb development; (**K**) Shoot multiplication in solid medium; (**L**,**M**) Bulb development and leaves growing in liquid medium; (**N**) Plant developed ready to acclimation in greenhouse. Bar = 1 cm.

The best *R*. *bifida* axillary shoot regeneration was obtained with medium 7 (Fig. 3C–H), followed by two media with two growth regulator compositions, one containing NAA 0.1 mg/L + BA 5 mg/L and the other with higher concentration of sucrose (60 g/L). Before the transfer to the liquid medium, the shoot formation took place with aggregates containing 3 to countless micro-plants per explant (Fig. 3K).

For bulbs growth (Fig. 3I), the micro-plants still attached to the explant were transferred to the multiplication medium (Fig. 3J,K), passing through different developmental stages, reaching the transfer stage in the liquid medium (Fig. 3L,M). This allowed the enlargement of bulb mass and leaf expansion of regenerated plants up to the time of acclimation in the greenhouse (Fig. 3N), once the bulb was completely formed.

After 45 days in the multiplication medium, the number of regenerated plants reached 38 plants/explant and 24 complete bulbs with growing roots developed for each explant grown on the Medium 7. This medium contains NAA and BA as growth regulator and was used in the initial phase plant regeneration.

### Characterization of the *RbCYP96T* and *RbN4OMT* genes

AmAl represent a large class of molecules present exclusively in this plant family. More than 300 different structures with a large range of biological activities have already been described. Although the initial part of the biosynthetic pathway has been described, most of the enzymes and the corresponding genes remain unknown^17^. The *N4OMT* and *CYP96T* gene sequences have only been identified in plants producing galanthamine. Molecules such as montanine have been well described for their biological activities however, molecular data date back from decades and the key genes of this pathway still need to be described to boost biotechnological and pharmaceutical studies.

The methyltransferase enzyme N4OMT is able to methylate norbelladine to form 4′-*O*-methylnorbelladine in *Narcissus*^27^. To identify the *R*. *bifida N4OMT* gene, we designed oligonucleotides based on the genomic sequence from close homologs of *N4OMT* in *Narcissus* (*NpN4OMT1* to *NpN4OMT4*; Online Resource, Supplementary Material Fig. S1). For this gene, the oligonucleotides included the ATG and stop codons. *R*. *bifida* cDNA fragment (744 bp) was cloned and sequenced from several clones providing a unique full-length *R*. *bifida N4OMT* cDNA sequence. Our study demonstrates that a single *N4OMT* gene is expressed in *R*. *bifida* bulbs. The putative *R*. *bifida N4OMT* gene coding sequence shows respectively 93, 92, 92, 92 and 93% identity with the coding sequences of the *NpN4OMT1* (KJ584561.1), *NpN4OMT2* (KJ584562.1), *NpN4OMT3* (KJ584563.1), *NpN4OMT4* (KJ584564.1) and *NpN4OMT5* (KJ584565.1) genes encoding the *N*. aff. *pseudonarcissus* N4OMT protein^27^; Online Resource, Fig. S1). The 239-amino acid *R*. *bifida* protein shows 92 to 93% identity to the *Pseudonarcissus* protein. Thus, the gene we cloned here represents the *R*. *bifida* Norbelladine 4′-*O*-Methyltransferase ortholog (*RbN4OMT;* GenBank: MH765575).

The CYP96T enzyme catalyzes the transformation of 4′-*O*-methylnorbelladine to noroxomaritidine, leading to the formation of noroxomaritidine derivatives, such as haemanthamine^18^ and montanine. To identify the *CYP96T* gene ortholog in *R*. *bifida*, we also aimed at designing oligonucleotides based on conserved regions of the *Pseudonarcissus CYP96T1* gene. The coding sequence of this gene was 1539 bp long and encoded a 513 amino acids protein^18^. However, the *N*. *pseudonarcissus* highly homologous sequences corresponding to CYP96T3 (AMO65743.1), CYP96T2 (AMO65742.1) and Noroxomaritidine synthase Cytochrome P450 96T1 (A0A140IL90.1) present in the Genebank did not allow defining conserved regions. Using genomic data from *Lycoris*, we found a partial genomic sequence homologous to the *Pseudonarcissus* CYP96T genes covering 415 bp of the 3′ part of the coding sequence. Using a combination of oligonucleotides specific of the *CYP96T* genes including the *Lycoris* sequence, we succeeded to amplify a *Rhodophiala* cDNA sequence covering 384 bp (166 aa) of the putative *R*. *bifida CYP96T* sequence. This sequence covered 25% of the 3′ part of the *Pseudonarcissus CYP96T1* coding sequence and showed 85% of nucleotide identity and 84% of identity (93% similarity) at the protein level relative to *Pseudonarcissus* CYP96T (AMO65743 ^18^) (Online Resource, Fig. S2). The partial sequence corresponding to a putative *para-para’*C-C phenol coupling cytochrome P450 in *R*. *bifida* was named *Rb*CYP96T (GenBank: MH765576).

### *RbCYP96T* and *RbN4OMT* gene expression in *R*. *bifida*

Studies initiated in the 80’s showed that alkaloids may influence plant growth as growth stimulators and regulators. However, the most important function of AmAl is the protection of plant cells from physical stresses such as UV-light, heat, pathogens and herbivores. AmAl can also help the plant to adapt to its local environment^38^. To determine the developmental factors affecting alkaloid production and accumulation we studied *RbCYP96T* and *RbN4OMT* transcripts accumulation in different organs and under different growing conditions. Gene expression analyses were performed on reverse transcribed RNA isolated from *R*. *bifida* roots, bulbs and leaves grown in greenhouse for wild plants or acclimated plants of *in vitro* regenerated plants, or from *in vitro* grown young plants.

**Figure 4:**
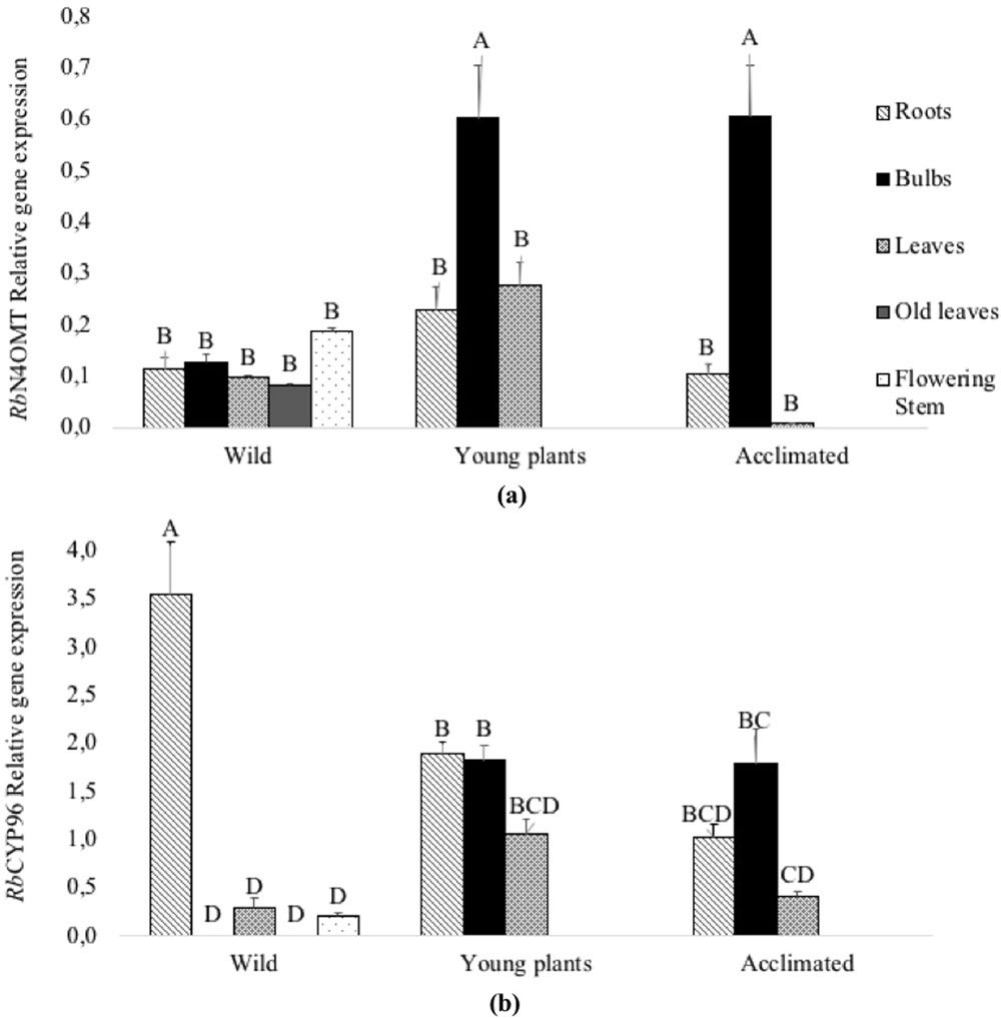
*RbN4OMT* and *RbCYP96T* relative gene expression in wild adult, young and acclimated plants. (**a**) *RbN4OMT* and (**b**) *RbCYP96T* relative transcript accumulations were normalized on *RbACTIN* gene expression and assessed in different plant organs and growing conditions in *R*. *bifida*. (**a**,**b**) Results represent means +/− SE of three biological repeats and two technical repeats. Letters indicate significant differences (Tukey test, *alpha* < 0.05).

Under the different growing condition tested, *RbN4OMT* was expressed in almost all tissues (Fig. 4a). The highest *RbN4OMT* gene expression level was found in bulbs of both young plants and acclimated plants. However, wild plants did not show significantly higher expression in bulbs. We believe that in the wild bulbs the expression remained basal due to the absence of cell division and vascular tissue formation when stem width growth is complete^39^. In the other organs tested, *RbN4OMT* gene expression was always lower relative to bulbs whatever the growing conditions. Almost no expression was detected in leaves of acclimated plants (Fig. 4). In order to obtain a better visualization of the *RbN4OMT* gene expression in *R*. *bifida* plants, the analysis was done using additional organs of the wild plants (old leaves and flowering stems, Fig. 4). This analysis indicates that this gene was similarly expressed in roots, bulbs, leaves, old leaves and flowering stems. These levels of expression detected in the wild plants may be correlated with the age of these plants and their developmental stages. All together these results suggest that the *RbN4OMT* gene was expressed in the growing tissues of the developing plants. During seedling growth and acclimation, the highest level of *RbN4OMT* expression might be linked to the methylation of Norbelladine (**9**) to form 4′-*O*-methylnorbelladine (**10**) in bulbs.

In *R*. *bifida*, the *RbCYP96T* gene was also expressed in the majority of the tissues and culture conditions analyzed (Fig. 4b). Maximum *RbCYP96T* gene expression was found in wild plant roots while in bulbs and old leaves, the expression was below the detection level. In young growing *in vitro* plants, the highest expression was detected in roots and bulbs followed by leaves. In acclimated plants the highest level of expression was detected in bulbs followed by roots and leaves.

This expression study shows that the *RbN4OMT*and *RbCYP96T*expression patterns are correlated in roots and bulbs of the young and acclimated plants and suggests production of montanine in these organs, in agreement with its accumulation in the roots and bulbs of adult plants.

### Montanine production in *R*. *bifida* organs

We wanted to correlate gene expression with AmAl production. First, we evaluated the linearity of our HPLC-based quantification method by constructing three linear curves on three different days with solutions containing montanine in seven different concentrations ranging from 120 to 2200 μg/mL. The linearity curves showed good linear correlation (R² = 0.999 and linear equation y = 13 036 × −371 135). These standard curves were used for AmAl quantification in our extracts.

Montanine quantification (Table 4) showed the highest production in bulbs and roots of wild plants (3.53 and 3.42 mg/g, respectively), followed by young plants bulbs (2.21 mg/g), young plants leaves (2.10 mg/g), acclimated roots and leaves (1.63 and 1.47 mg/g, respectively) plus wild plant leaves (1.42 mg/g). The lowest levels of montanine were detected in young plant roots (1.19 mg/g) and in the old leaves (0.30 mg/g).

**Table 4:** Montanine content (mg/g) found in the organs of *Rhodophiala bifida* plants cultured in different growth conditions.

Organ	Growth conditions		
	Young plants	Acclimated	WT
Roots	1.19 D	1.63 BCD	3.42 A
Bulbs	2.21 B	1.26 D	3.53 A
Leaves	2.10 BC	1.47 BCD	1.42 CD
Old leaves	na	na	0.30 E
Stem	na	na	1.25 D

*Means not followed by the same letter differ from each other by the Tukey test, at 5% error probability. Young plants: young growing *in vitro* plants; Acclimated: greenhouse acclimated regenerated plants; WT: greenhouse grown wild plants; nd: not analyzed.

In wild plant tissues (Table 4) the amount of montanine in the bulbs and the roots (3.53 and 3.42 mg/g, respectively), was about 2.5 times higher than in the leaves and the flowering stems (1.42 and 1.25 mg/g). The lowest yield was found in old leaves (0.30 mg/g) almost 12 times lower than the amount produced by bulbs.

## Discussion

In this study we have shown that the GC-MS analysis allowed the detection of 21 different compounds and we confirmed that montanine was the most abundant AmAl detected in the different *R*. *bifida* organs. Some of the detected compounds represented intermediates of the montanine biosynthesis pathway.

Amaryllidaceae regeneration was mainly described for *Narcissus confusus* and *Leucojum aestivum*^40–44^. In studies using *L*. *aestivum*, embryogenesis occurred in presence of dicamba and picloram, in 30 and 100% of the explants, respectively. In *L*. *aestivum*, 2,4-D promoted greater callus proliferation and somatic embryogenesis by contrast to other auxins^42^. For *N. confusus*, the regeneration was obtained through meristematic callus formation using 3% sucrose with BA and kinetin. This resulted in the formation of globular calli that turned green and developed aggregates of young shoots, similarly to *L*. *aestivum*, in which it allowed growth of embryogenic nodular calli^40,41^. The largest number of regenerated *L*. *aestivum* plants^42^ was obtained using BA and meta-topolin and a combination of meta-topolin and thidiazuron to form complete plants. In this study^42^, plant formation from 1 g of embryos was the best in media enriched with meta-topolin or benzyladenine, however the use of thidiazuron and meta-topolin led to the highest quantity of normally developed plants^44^. In a study using *Narcissus* cultivars^43^ addition of sucrose increased the explant survival and callus formation, and resulted in better organogenesis.

We present here a complete protocol for regeneration using *R*. *bifida* twin scales. The medium containing NAA (0.1 mg/L) and BA (0.5 mg/L) can induce shoot formation and together with a multiplication in solid medium supplemented with activated charcoal, results in the generation of plant clumps. When transferred to liquid medium these plant clumps develop bulbs with roots in absence of any regulators. Also, these micro-plants can be transferred to the greenhouse where they develop normally. We also present the necessary procedure to transfer *in vitro* plants to soil in a greenhouse. These conditions can be adopted for future large-scale productions.

In order to initiate the molecular characterization of the AmAl biosynthetic genes we identified the full-length *RbN4OMT* and the partial Rb*CYP96T* P450 cDNA sequences. Also we provide functional primers for the Amaryllidaceae *ACTIN* gene suitable for *R*. *bifida* gene expression studies. We showed that the expression of the *RbN4OMT* gene was rather low in wild plants with similar expression between the organs evaluated. In these plants, the level of montanine was equivalent and relatively high in the root and the bulbs but lower in the old leaves. In agreement with the level of expression observed in bulbs of young growing *in vitro* or acclimated plants, we can hypothesize that the production of alkaloids takes place earlier during plant development. Similarly, in *Lycoris sprengeri* bulbs, the expression of genes involved in starch biosynthesis decreased as the bulb diameter increased^45^. In wild *N*. *pseudonarcissus* collected during the flowering season, the highest *N4OMT* expression was also detected in bulbs, followed by inflorescences (intermediate results) and leaves (close to zero)^27^. It should also be noticed that the molecules present in an organ might have been synthetized and transported towards other organs as observed in *Narcissus* Carlton^46^.

The *RbCYP96T* gene was expressed at high level in the wild roots but was below detection level in the bulbs and old leaves despite the high content of montanine in these organs. Since the bulb is an underground storage organ, it is possible that biosynthesis of the alkaloids occurs earlier during bulb development in order to protect carbohydrate resources of the plant against herbivores and microorganisms^47,48^. Alternatively, a second *CYP96T* gene might be expressed specifically in the bulbs of this plant and was not detected in our experiments. The young growing *in vitro* and acclimated samples presented intermediate expression levels for the *RbCYP96T* gene, principally in roots and bulbs, in agreement with their intermediate alkaloid accumulation.

Thus, altogether the expression study of these two genes correlates with a synthesis starting in developing organs and with an alkaloid accumulation during growth resulting in the higher montanine content observed in roots and bulbs of this plant.

In a study carried out with *N*. *pseudonarcissus* L. cv. Carlton^46^, production of alkaloids in bulbs, roots and leaves was the highest before flowering and bulbs had the lowest concentrations of the compounds galanthamine, haemanthamine and narciclasine. In *Lycoris aurea*, alkaloid content increased during the growing season and the galanthamine content was the lowest in leaves and the highest in bulbs. For this plant, around 99.5% of galanthamine was detected in bulbs and roots, with the highest level identified in roots and bulbs at the time of leaves withering in autumn^49^. In *Crinum macowanii*, bulbs had also the highest alkaloid content, followed by roots, flower stalks and leaves^50^. These data show certain similarity to our results in which the highest montanine content was found in the adult roots and bulbs. Furthermore, in *Leucojum aestivum*, the alkaloid biosynthesis and content depend of the latitude as well as of soil fertility level^51,52^. Our results also suggest that *R*. *bifida* alkaloid accumulation depends on the plant developmental stage.

Our work provides new insights regarding the AmAl biosynthesis regulation and represents a first step towards a better understanding of the molecular characterization of the montanine biosynthetic genes in *R*. *bifida*, awaiting sequencing of this plant genome. Our gene expression study using several plant organs and various growing conditions represents a founding study for future research aiming to know how *R*. *bifida* gene expression and alkaloid production can be correlated and improved to increase the production of this important pharmaceutical compound using *R*. *bifida*.

## Methods

### Plant material and *in vitro* culture

*Rhodophiala bifida* plants were collected in Pelotas (RS-Brazil) (Latitude: −31.7719; Longitude: −52.3425; err: ±51462 WGS84) in July 2015. The bulbs were separated, washed under running water, pre-disinfected in 2% sodium hypochlorite for 3 hours, washed in sterile water and dried at 23 °C for 24 h. Bulbs were stored at room temperature and before transfer to *in vitro* culture or greenhouse, they were kept at 10 °C and low humidity. The seeds of *R*. *bifida* var. Spathacea were purchased commercially (Jelitto®, Germany).

For greenhouse culture, a low organic mix (~1:3 organic: inorganic) of soil: sand: perlite (25: 40: 35%) was used. Plants were watered twice a week and nutritive solution was applied each ten days. Plants were grown without incident light.

Seed sterilization was performed in ethanol 70% for 1 min, in sodium hypochlorite 3% with tween 20 (two droplets per 100 mL) for 20 min. Seeds were washed 3 times in sterile water. For germination, seeds were placed on agar plates (10 g/L) at 20 °C (day) and 16 °C (night) for ten days in the dark. For young plants, seedlings were grown individually in glass tubes on ½ MS medium, sucrose 15 g/L, *Plant Preservative Mixture*^*TM*^ (PPM®, Plant Cell Technology, Washington, DC, US) 200 μL/L and agar 7 g/L. The medium was renewed every three weeks.

Outer dark layers of *in vitro* cultured (IC) bulbs were removed before sterilization in 70% ethanol for 1 min, followed by different schemes of disinfection as indicated in the text: I) Sodium hypochlorite 2% (commercial bleach) during 30 min, II) heating water to 54 °C during 1 h and then transfer to hypochlorite 2% for 1 h^36^ and III) pre-disinfection in 2% sodium hypochlorite for 3 hours, washing in distilled and autoclaved water and oven drying at 23 °C for 24 h + sodium hypochlorite 8% (commercial bleach) during 30 min. Bulbs were washed three times in sterile water, dried on filter paper for 30 min and sectioned into 1 × 1.5 cm fragments for *in vitro* culture.

Shoot induction from *R*. *bifida* bulbous explants was made on medium for Amaryllidaceae^53^, containing the macronutrients of the MS medium (Duchefa®, Haarlem, The Netherlands) plus inositol 100 mg/L, nicotinic acid 0.5 mg/L, thiamine 0.1 mg/L, pyridoxine 0.5 mg/L, casein 1 g/L, glycine 2 mg/L, phytagel 2 g/L, sucrose 30 g/L (all chemicals purchased from Sigma, St. Louis, MO, USA) and PPM at 0.2 g/L, pH 5.5 and autoclaved at 121 °C, at 1 atm for 20 min. Growth Regulators (GR) were sterilized by filtration (Millipore® 22 μm; Merck KGaA, Darmstadt, Germany) and added after autoclaving (121 °C for 20 min) as follows: Medium 1 or control: sucrose 30 g/L; Medium 2: sucrose 60 g/L; Medium 3: sucrose 90 g/L; Medium 4: NAA 5 mg/L + KIN 0.5 mg/L + BA 1 mg/L; Medium 5: 2,4-D 1 mg 1 mg/L + BA 1 mg/L; Medium 6: NAA 2 mg/L + BA 4 mg/L; Medium 7: NAA 0.1 mg/L + BA 0.5 mg/L; Medium 8: NAA 1 mg/L + BA 2 mg/L; Medium 9 NAA 0.1 mg/L + BA 5 mg/L and Medium 10: NAA 2.5 mg/L + KIN 0.5 mg/L (all the GR from Sigma, St. Louis, MO, USA). The explants were kept in a growth chamber for 60 days and evaluated for percentage of callus induction, shoots and bulbs per treatment.

After shoot growth, they were transferred to flasks containing culture medium for multiplication^53^ with some modifications. This medium consisted in MS medium base with the addition of inositol 100 mg/L, nicotinic acid 0.5 mg/L, thiamine 0.5 mg/L, pyridoxine 0.5 mg/L, glycine 1 mg/L, agar 7 g/L, activated charcoal 5 g/L (Sigma, St. Louis, MO, USA), sucrose 30 g/L, and PPM at 0.2 g/L, pH 5.5. After the multiplication of the shoots, the plants were relocated to the same modified MS medium but liquid and without charcoal. The media containing the micro-plants were renewed every 3 weeks. Samples were collected after 30, 60 and 90 days of growth. After 90 days in culture, the *in vitro* plants were acclimated to the greenhouse and after eight and six months the wild and acclimated plants, respectively, were harvested.

For alkaloids extraction, the plant material was oven dried at ± 40 °C and grinded. For gene expression analysis by Quantitative Real Time-PCR, the samples were frozen in liquid nitrogen. Chemical and molecular analyses were carried out on roots, bulbs and leaf organs.

### Gene expression analysis

Total RNA extractions and qRT-PCR reactions were performed as described by Berrabah and collaborators^54^.

Primer efficiencies were calculated with LinReg PCR: Analysis of Real-Time PCR Data, version 11.1. *RbN4OMT* and *RbCYP96T* gene expression were normalized, using *RbACTIN* as reference gene. These primers specifically designed to monitor *R*. *bifida ACTIN* gene expression were TCCATCATGAAGTGTGATGTTGATAT as forward primer and CCTCCAATCCAGACACTGTACTT as reverse primer.

To measure the expression of the *RbN4OMT* and *RbCYP96T* genes, the primers used were CTGCTTGGAATTCAAGCCCGA as forward primer and ATGCCCCTGGACCATCCGAA as reverse primer. For the *RbN4OMT* CGTCAGGGTTGGAGGAGCGAT (forward primer) and ATCGCTCCTCCAACCCTGACG (reverse primer) were used.

### *R*. *bifida* cDNA Cloning

All the primers were designed based on *CYP96T1* and *N4OMT* Amaryllidaceae sequences conserved at the amino acids and nucleotides level in publicly available sequences (NCBI; GenBank^55^). Sequence comparisons were done with Blast search (http://www.ncbi.nlm.nih.gov/BLAST/) using sequences from the Amaryllidaceae family, *OMT* and *CYP96T* as criteria. For the *N4OMT* the forward primer was ATGGGTGCTAGCATAGATGATTAT and the reverse primer TCAATAAAGACGTCGGCAAATAGT. For the *CYP96T* the forward primer was CCATGGCCACTTCTTCTTCAGCATG and the reversed primer CCTCACATGACTGATCTCTTTCTAA. PCRs were done using Phusion High-Fidelity DNA polymerase (Thermo Scientific, USA) in a protocol containing 1 pre-incubation cycle (95 °C, 5 min) and 35 amplification cycles [(denaturation: 95 °C, 30 s), (hybridization: 55 °C, 30 s), (elongation: 72 °C, 1 min)].

The PCR products were cloned in pGEM®-T Easy Vector (Promega, USA) and introduced in One Shot® TOP10 Chemically Competent *E*. *coli* for plasmid amplification (Promega, USA).

Plasmid inserts were sequenced using Eurofins Genomics facilities (Ebersberg, Germany).

### Alkaloid extraction and HPLC analysis

The extraction of total alkaloids was performed according to^56^ with small modifications. We used 100 mg of plant material and the extraction was initiated with 2% sulfuric acid (v/v) on an ultrasound bath for 4 hours. The samples were gravity filtered and the supernatant washed with ethyl ether (3 × 100 mL). The aqueous fraction was basified with 25% ammonium hydroxide (v/v) until pH 9. This basic aqueous solution was partitioned with ethyl acetate (3 × 100 mL). The organic residue was filtered on anhydrous sodium sulfate and the volume was reduced to the residue in a rotatory evaporator. The alkaloid rich residue was resuspended in methanol 2 mL, filtered through a 0.45 μm membrane (Millipore®) and analyzed.

Liquid chromatographic analyzes were performed on a Waters Alliance e2695 with a diode arrangement detector (PDAWaters 2998), and software Empower 3 HPLC (Waters^®^) for data acquisition and treatment. The chromatographic column employed was reverse phase (Synergi Polar Phenomenex^®^, 80 Å, 4 μm, 250 × 4.60 mm) and coupled to a reversed-phase pre-column (Security Guard Cartridges™ Fusion Phenomenex^®^; 4.0 × 3.0 mm). The elution system used was composed by trifluoroacetic acid (TFA) 0.01% (mobile phase A, v/v) and acetonitrile: TFA 0.08% (mobile phase B, v/v). The flow rate used was 0.5 mL min^−1^, temperature for the sample compartment 20 °C and the gradient start with solvent A at 85% (0–13 min), 82% (2 min); 79% (2 min), 77% (2 min); 100% of solvent B (hold for 2 min), 85% of solvent A (for the next 2 minutes), totaling 22 minutes of analysis using the wavelength of 290 nm for the montanine quantification.

### Alkaloid identification by GC-MS

About 100 mg of each sample were dissolved in 100 µL of methanol and injected directly into the GC-MS apparatus (Agilent Tecnologies 6890 N coupled with MSD5975 inert XL) operating in the EI mode at 70 eV. A Sapiens-X5 MS column (30 m × 0.25 mm i.d., film thickness 0.25 µm) was used. The temperature gradient performed was the following: 2 min at 100 °C, 100–180 °C at 15 °C/min, 180–300 °C at 5 °C/min and 10 min hold at 300 °C. The injector and detector temperatures were 250 °C and 280 °C, respectively, and the flow-rate of carrier gas (He) was 1 mL/min. A split ratio of 1:10 was applied and the injection volume was 1 µL.

### Statistical analysis

For the chemical analysis, we used at least three biological replicates for all of the samples and two technical replicates. For the qRT-PCR, three biological replicates and two technical replicates were done. The data were analyzed using analysis of variance (ANOVA) and means tests were compared by Tukey test, with a 5% level of significance (*p* < 0.05).

## Supplementary information

Supplementary information
