# Author Correction: Amaryllidaceae alkaloids: identification and partial characterization of montanine production in *Rhodophiala bifida* plant

**DOI:** 10.1038/s41598-019-50830-9

**Published:** 2019-10-29

**Authors:** Andressa Reis, Kevin Magne, Sophie Massot, Luciana R. Tallini, Marina Scopel, Jaume Bastida, Pascal Ratet, José A. S. Zuanazzi

**Affiliations:** 1 Laboratory of Pharmacognosy, Department of Raw Material Production, Federal University of Rio Grande do Sul, 90610-000 Porto Alegre, UFRGS Brazil; 2 Institute of Plant Sciences Paris-Saclay IPS2, CNRS, INRA, Université Paris-Sud, Université Evry, Université Paris-Saclay, Bâtiment 630, 91405 Orsay, France; 3 Institute of Plant Sciences Paris-Saclay IPS2, Paris Diderot, Sorbonne Paris-Cité, Bâtiment 630, 91405 Orsay, France; 4 Natural Products Group, Faculty of Pharmacy, University of Barcelona, Av. Joan XXIII, 27-31, 08028 Barcelona, Spain; 5 Present Address: Laboratory of Molecular Biology, Department of Plant Sciences, Wageningen University & Research, 6708 PB Wageningen, The Netherlands

Correction to: *Scientific Reports* 10.1038/s41598-019-44746-7, published online 11 June 2019

The reference lists were omitted from the Supplementary Information file originally published with this Article. The Supplementary Information file has now been replaced.

In addition, this Article contained an error in the Introduction.

“Studies with *R*. *bifida* plants^15,20^ indicate that 11-hydroxyvittatine (**25**) (haemanthamine ring system) is formed by the addition of a hydroxyl group to the vittatine (**13**).”

now reads:

“Studies with *R*. *bifida* plants^15,20^ indicate that 11-hydroxyvittatine (**26**) (haemanthamine ring system) is formed by the addition of a hydroxyl group to the vittatine (**13**).”

This error has now been corrected in the HTML and PDF versions of the Article.

