# Supplementary material for: Amaryllidaceae alkaloids: identification and partial characterization of montanine production in Rhodophiala bifida plant
Source: Sci Rep. 2019 Jun 11;9:8471. doi: 10.1038/s41598-019-44746-7 (PMC6559983; doi:10.1038/s41598-019-44746-7)
Supplement: Supplementary file 1 — Supplementary information [file 41598_2019_44746_MOESM1_ESM.pdf]

Supplementary information

Amaryllidaceae alkaloids: identification and partial characterization of montanine production in *Rhodophiala bifida* plant

Andressa Reis[1], Kevin Magne[$2,3], Sophie Massot[2,3], Luciana R. Tallini[1,4], Marina Scopel[1], Jaume Bastida[4], Pascal Ratet*[2,3], José A. S. Zuanazzi[1].

[1]Laboratory of Pharmacognosy, Department of Raw Material Production – Federal University of Rio Grande do Sul, Porto Alegre - UFRGS, 90610-000, Brazil.

[2]Institute of Plant Sciences Paris-Saclay IPS2, CNRS, INRA, Université Paris-Sud, Université Evry, Université Paris-Saclay, Bâtiment 630, 91405 Orsay, France

[3]Institute of Plant Sciences Paris-Saclay IPS2, Paris Diderot, Sorbonne Paris-Cité, Bâtiment 630, 91405 Orsay, France.

[4]Natural Products Group, Faculty of Pharmacy, University of Barcelona, Av. Joan XXIII, 27-31, 08028-Barcelona, Spain

[$]Actual address: Laboratory of Molecular Biology, Department of Plant Sciences, Wageningen University & Research, 6708 PB, Wageningen, The Netherlands

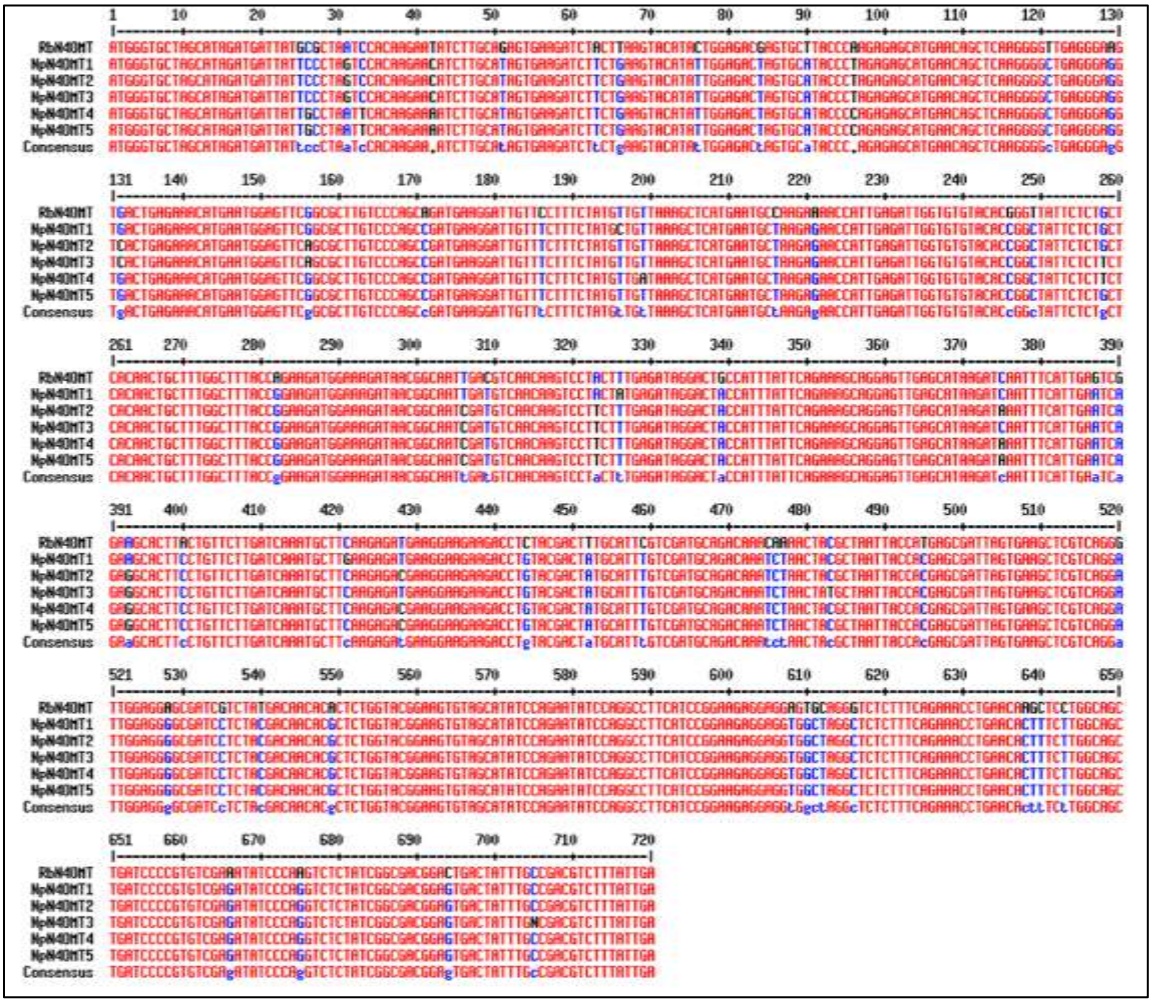

Figure S1: N4OMT sequences comparison: Multi-alignment analysis (Corpet F, 1988) showing high identity (in red) between the nucleotide sequence found in our study, *Rb*N4OMT, to N4OMT reported in *Narcissus*aff. *pseudonarcissus* MK-2014 sequences (Kilgore et al., 2014) sharing a similarity of 93, 92, 92, 92 and 93% with *Np*N4OMT1, *Np*N4OMT2, *Np*N4OMT3, *Np*N4OMT4 and *Np*N4OMT5.

<u>References</u>:

Corpet, F. Multiple sequence alignment with hierarchical clustering. Nucl. Acids Res., 16 (22), 10881-10890 (1988).

Kilgore, M. B. *et al.* Cloning and characterization of a norbelladine 4′-O-methyltransferase involved in the biosynthesis of the Alzheimer's drug galanthamine in *Narcissus* sp. aff. *pseudonarcissus*. *PLoS One* **9,** 1–11 (2014).

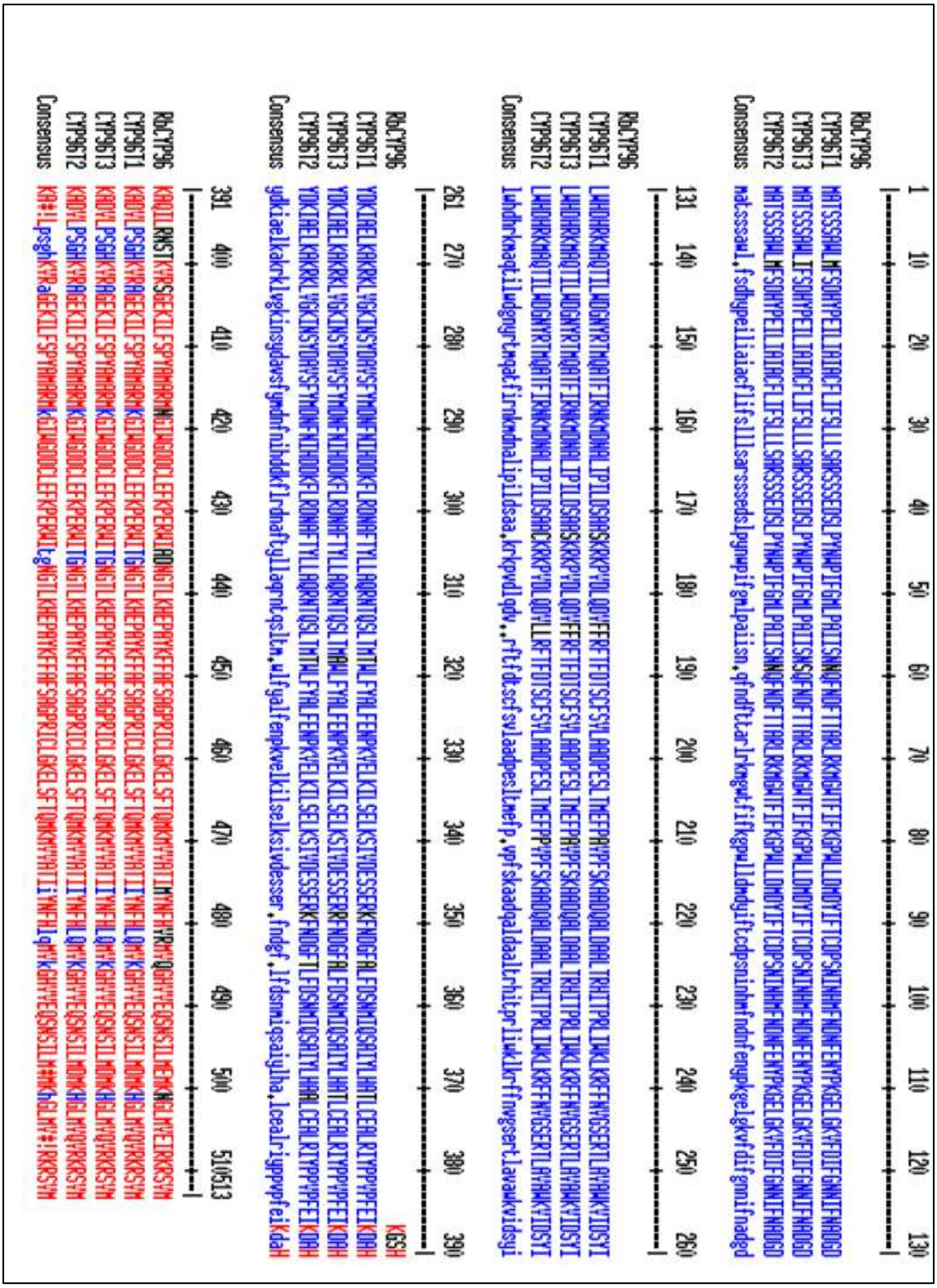

Figure S2: CYP96 sequence comparison: Multi-alignment analysis showing high identity (in red) between the protein sequence found in *R. bifida*, *Rb*CYP96, when correlated with *N.*aff. p*seudonarcissus* CYP96 protein

sequences (Kilgore et al., 2016). The nucleotide sequence shows 84% identity with CYP96T3, CYP96T2 and Noroxomaritidine synthase Cytochrome P450 96T1.

Reference:

Kilgore, M. B., Augustin, M. M., May, G. D., Crow, J. A. & Kutchan, T. M. CYP96T1 of *Narcissus* sp. aff. *pseudonarcissus* catalyzes formation of the *Para-para*' C-C phenol couple in the Amaryllidaceae alkaloids. *Front. Plant Sci.***7,** 1–16 (2016).

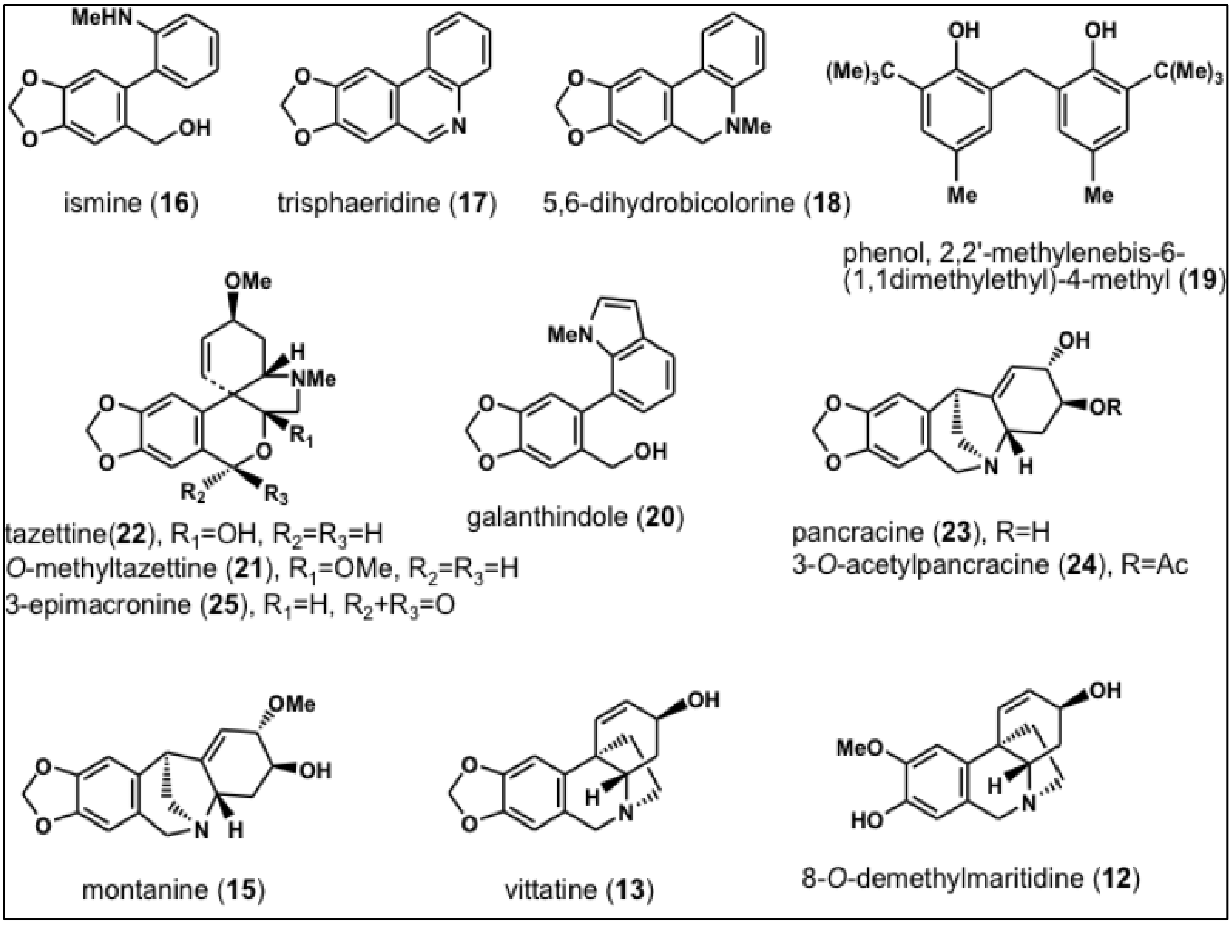

Figure S3 Alkaloids identified in *R. bifida* wild plants.
